# Supplementary material for: An All-Solid-State Flexible Supercapacitor Based on MXene/MSA Ionogel and Polyaniline Electrode with Wide Temperature Range, High Stability, and High Energy Density
Source: Molecules. 2023 Feb 6;28(4):1554. doi: 10.3390/molecules28041554 (PMC9960789; doi:10.3390/molecules28041554)
Supplement: Supplementary file 1 [file molecules-28-01554-s001.zip › molecules-2145705-supplementary.pdf]

An all-solid-state flexible supercapacitor based on MXene/MSA ionogel and polyaniline electrode with wide temperature range, high stability and high energy density

Feng Pan[1], Shuang Wang [1,2*], Zhipeng Yong [1], Xiaodong Wang [1], Chenglong Li[1], Dan Liang [1], Xiaorui Wang [1], Han Sun[1], Yinghe Cui [1], Zhe Wang [1,2*]

[1] College of Chemical Engineering, Changchun University of Technology, Changchun, 130012, People's Republic of China

[2] Advanced Institute of Materials Science, Changchun University of Technology, Changchun, 130012, People's Republic of China

Corresponding author E-mail:

wangshuang_ccut@163.com (S.W.); wzccut@126.com (Z.W.)

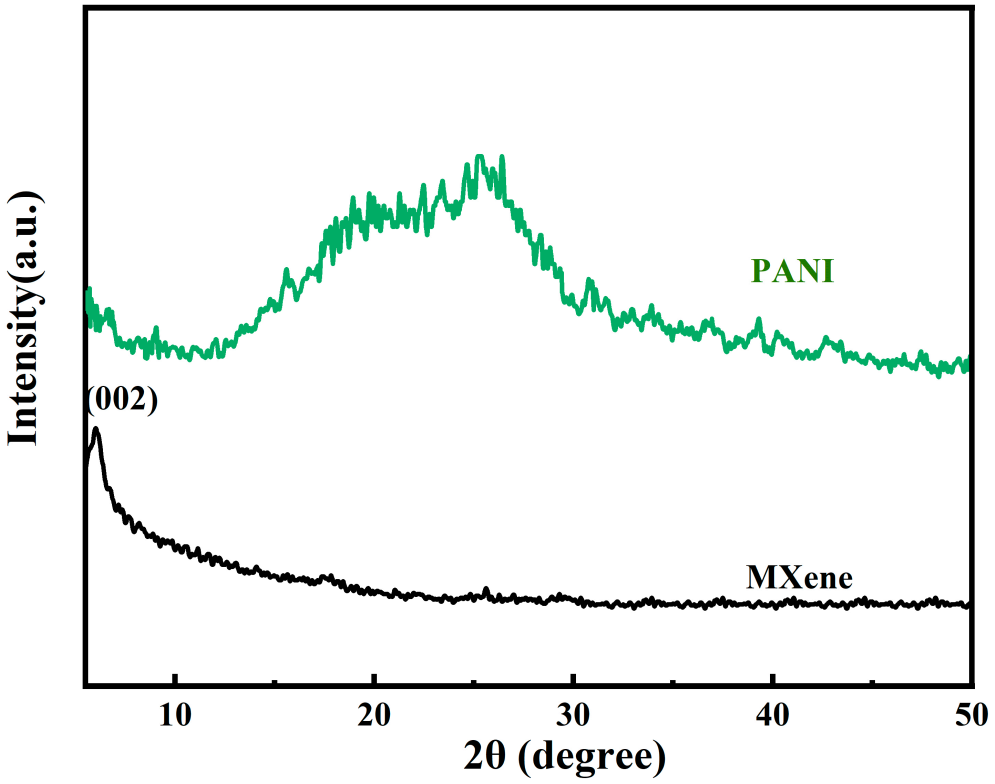

Figure S1. XRD images of PANI and MXene.

For PANI samples, prominent peaks at scattering angles of 2θ = ~15° and~25° were observed. These peaks are attributed to the periodicity of the repeat unit of PANI chain and periodicity parallel to the polymer chain backbone. For MXene samples, prominent peak at scattering angles of 2θ = ~2° was observed. These peaks validate the

success of PANI and MXene.

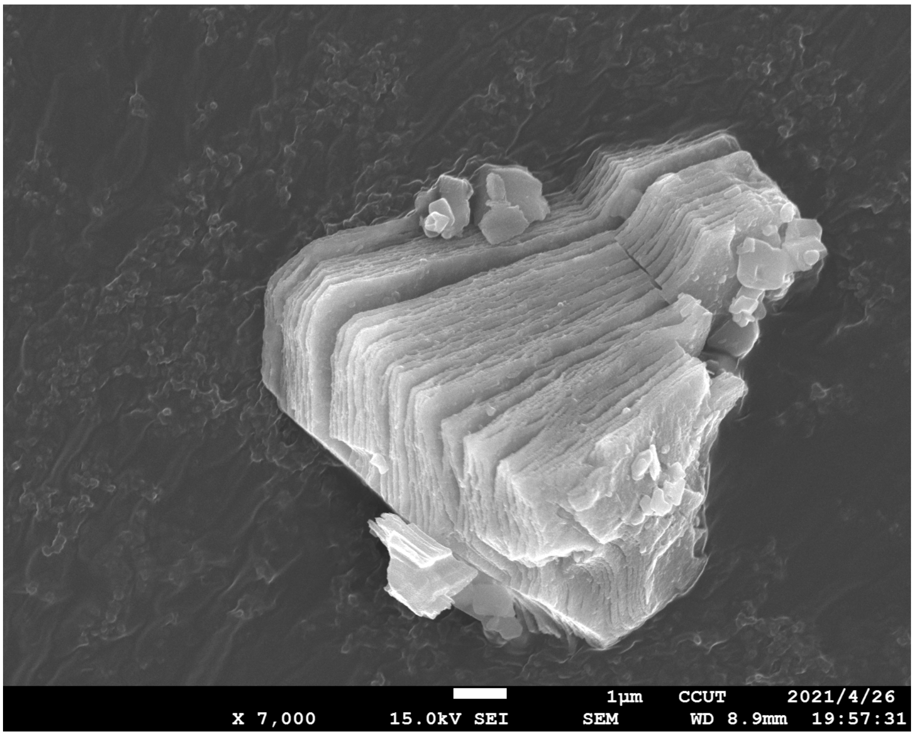

Figure S2. SEM images of MXene.

The MXene image is in the shape of a standard stacked accordion, confirming its successful synthesis.

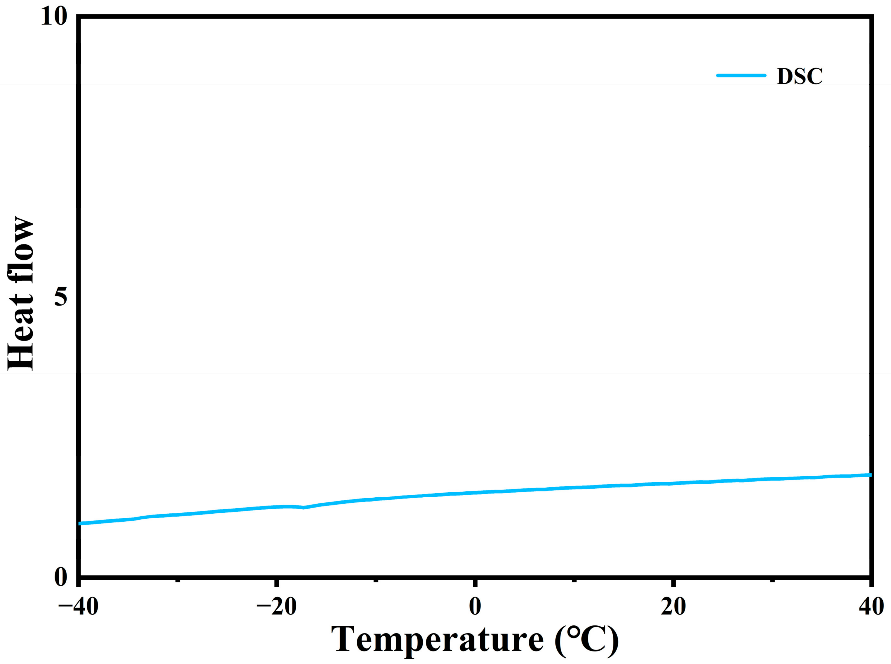

Figure S3. DSC images of PAIM.

In DSC test, there is no freezing point of PAIM from -40 °C to 40°C. In fact, we

can not freeze-dry PAIM with freeze-dryer, it also shows that the ionogels with MSA as solvents have a broader research and application prospects. This is in line with previous research[43].
